# Inflammatory Response of THP1 and U937 Cells: The RNAseq Approach

**DOI:** 10.3390/cells13242062

**Published:** 2024-12-13

**Authors:** Layla Panahipour, Chiara Micucci, Reinhard Gruber

**Affiliations:** 1 Department of Oral Biology, University Clinic of Dentistry, Medical University of Vienna, 1090 Vienna, Austria; layla.panahipour@meduniwien.ac.at (L.P.); chiara.micucci@studenti.unipr.it (C.M.); 2 Department of Periodontology, School of Dental Medicine, University of Bern, 3010 Bern, Switzerland; 3 Austrian Cluster for Tissue Regeneration, 1200 Vienna, Austria

**Keywords:** THP1, U937, RNAseq, inflammation, bioassay, LPS, IFNG, macrophages

## Abstract

THP1 and U937 are monocytic cell lines that are common bioassays to reflect monocyte and macrophage activities in inflammation research. However, THP-1 is a human monocytic leukemia cell line, and U937 originates from pleural effusion of histiocytic lymphoma; thus, even though they serve as bioassay in inflammation research, their response to agonists is not identical. Consequently, there has yet to be a consensus about the panel of strongly regulated genes in THP1 and U937 cells representing the inflammatory response to LPS and IFNG. Therefore, we have performed an RNAseq of THP1 and U937 exposed to LPS and IFNG to identify the most sensitive genes and the unique properties of each individual cell line. When applying a highly stringent threshold, we could identify 43, 8 up and 94, 103 down-regulated genes in THP1 and U937 cells, respectively. In THP1 cells, among the most strongly up-regulated genes are *CCL1*, *CXCL2*, *CXCL3*, *IL1A*, *IL1B*, *IL6*, and *PTGES*. In U937 cells, the strongest up-regulated genes include *CSF2*, *CSF3*, *CXCL2*, *CXCL5*, *CXCL6*, *IL1A*, *IL19*, *IL36G*, *IL6*, *ITGA1*, *ITGA2*, and *PTGS2*. Even though THP1 is considerably less responsive than U937, there are genes commonly upregulated by LPS and IFNG, including the *CCL1*, *CCL3*, *CCL20*, *CXCL2*, *CXCL3*, *CXCL8*, as well as *IL1A*, *IL1B*, *IL23A*, *IL6*, and genes of prostaglandin synthesis *PTGES* and *PTGS2*. Downregulated genes are limited to *NRGN* and *CD36*. This head-to-head comparison revealed that THP1 is less responsive than U937 cells to LPS and IFNG and identified a panel of highly regulated genes that can be applied in bioassays in inflammation research. Our data further propose bulk RNAseq as a standard method in bioassay research.

## 1. Introduction

Inflammation, when transient, is an evolutionally conserved process that is an essential requirement of innate immunity and signals the need for repair in wound healing [1] and bone regeneration [2]. On the other hand, chronic inflammation is a pathological process where resolution is impaired, and catabolic events accumulate and cause tissue destruction, exemplified by rheumatoid arthritis, Crohn’s disease, and periodontitis, to name a few [3,4,5]. Understanding the drivers and underlying molecular mechanisms of inflammation and developing strategies supporting its resolution are of fundamental importance in inflammation research throughout all disciplines of medicine [5,6]. Our progress in inflammation research is partially based on bioassays to simulate a clinically relevant inflammatory response. In vitro bioassays are, therefore, valuable tools to study the inflammatory response of potential target cells to virulence factors and other drivers of inflammation.

Among the professional inflammatory cells are monocytes that are recruited during infection and inflammation and can differentiate to become more specialized macrophages [7] or are ready to be activated on-site [8], apart from other functions in tissue development, homeostasis, and repair [9]. For research purposes, human monocytes can be isolated from blood and enriched by adhesion [10] or purified by their surface markers, including CD14 [11]. Human primary macrophages are highly sensitive to conserved molecular structures of invading pathogens detected by a series of Toll-like receptors and other pathogen recognition receptors. LPS from *E. coli (Escherichia coli)* is the classic example of initiating a macrophage-based inflammatory response in vitro. LPS is a pathogen-associated molecular pattern, conserved within a class of microbes that provokes an inflammatory response via Toll-like receptors. Interferon gamma (IFNG) is another potent inflammatory mediator produced by lymphocytes in disease and infections that even augments the Toll-like receptors inflammatory response [12]. LPS, in combination with IFNG, is, therefore, ideal to provoke a robust inflammatory response of macrophages [13].

Blood is a rich source of monocytes; however, cell isolation is time-consuming, costly, and inconvenient for the donor [10,14]. Moreover, the donor variation has to be considered. Even though cell lines cannot entirely reflect the response of a primary monocyte to LPS and IFNG, THP1 and U937 are widely used in inflammation research. THP1 and U937 were originally isolated from the peripheral blood of an acute monocytic leukemia patient [15] and from the pleural effusion of a patient with histiocytic lymphoma [16], respectively. Both cell lines, THP1 [17,18] and U937 [19,20] are widely used to study the inflammatory response when exposed to LPS in combination with IFNG in vitro. Considering their everyday use and the heterogeneity of their origin, comparing the two cell lines for their in vitro response was performed [21,22], which was further considered in other studies by secondary intension [23,24]. Additionally, selecting target genes regulated by LPS and IFNG was performed arbitrarily.

Target genes increasingly expressed by THP1 cells exposed to LPS and IFNG involve inflammatory mediators, including *CCL2*, *CXCL8*, *CXCL10*, *CCR7*, *IL1β*, *IL6*, *IL10*, *TNF*, and *PTGS2* [25,26]. For U937 cells, a comparable panel of genes was implemented, including *CCL2*, *IL1β*, *IL6*, *MMP2*, *MMP9*, and *PTGS2* [27]. These gene panels serve the purpose of monitoring an inflammatory response and its modulation by other compounds. Even though gene panels are reliable in reflecting the cell response to LPS and IFNG, other potential inflammatory genes, apart from the mentioned cytokines, chemokines, prostaglandins, and proteases will complete the spectrum of candidate genes to establish a bioassay. These candidate genes might include genes that go beyond the autocrine and paracrine function in THP1 and U937 when exposed to LPS and IFNG.

The aim of the present study was, therefore, to extend the effort of previous research to compare the response of THP1 and U937 cells [21,22] and also validate the current list of genes serving as markers of an inflammatory response [25,26,27]; we have performed an RNAseq screening approach. This RNAseq strategy allowed us to identify the strongly regulated genes of the two cell lines in familiar and novel genes representative of either THP1 or U937 cells, respectively.

## 2. Materials and Methods

### 2.1. THP1 and U937 Cells

The human monocytic cell lines THP1 and U937 were obtained from ATCC (TIB-202 and CRL-1593.2) and cultured in RPMI 1640 medium (Gibco Life Technologies, Carlsbad, CA, USA) supplemented with 10% FCS (Bio&Sell GmbH, Nuremberg, Germany) and 1% antibiotics in a humidified incubator at 37 °C with 5% CO_2_. Both cell lines were seeded into 24-well plates and exposed to 10 ng/mL phorbol-12-myristate-13-acetate (PMA, Sigma Aldrich, St. Louis, MO, USA) for 48 h for their differentiation into macrophages. Differentiated THP1 and U937 cells were then exposed to 50 ng/mL IFNG (ProSpec, Ness-Ziona, Israel) and 100 ng/mL LPS from *Escherichia coli* 0111: B41 (Sigma Aldrich, St. Louis, MO, USA) for 24 h before RNA extraction.

### 2.2. Total RNA Isolation, Sequencing, and Data Analysis

Total RNA was isolated with the GeneMATRIX Universal RNA purification kit (EUR_X_, Gdańsk, Poland) with DNAse digestion (Thermo Fischer, Waltham, MA, USA). Sequencing libraries from total RNA were prepared at the Core Facility Genomics, Medical University of Vienna, using the QuantSeq 3′ FWD protocol version 2 with unique dual indices (Lexogen GmbH, Vienna, Austria). Fifteen PCR cycles were used for library prep, as determined by qPCR according to the library prep manual. Libraries were QC-checked on a Bioanalyzer 2100 (Agilent Technologies, Santa Clara, CA, USA) using a high-sensitivity DNA Kit for correct insert size and quantified using Qubit dsDNA HS Assay (Invitrogen, Waltham, MA). Pooled libraries were sequenced on a P2 flowcell on a NextSeq2000 instrument (Illumina, San Diego, CA, USA) in 1 × 75 bp single-end sequencing mode. On average, 7 million reads per sample were generated. Reads in fastq format were generated using the Illumina bcl2fastq command line tool (v2.19.1.403) and the Lexogen idemux tool for optimal demultiplexing of long unique dual indices. Reads were trimmed and filtered using cutadapt version 2.8 to trim polyA tails, remove reads with N’s, and trim bases with a quality of less than 30 from the 3′ ends of the reads [28]. On average, 5 million reads were left after this procedure. Trimmed reads in fastq format were aligned to the human reference genome version GRCh38 with Gencode 29 annotations using STAR aligner [29] version 2.6.1a in 2-pass mode. STAR counted raw reads per gene. Differential gene expression was calculated using DESeq2 [30] version 1.22.2.

### 2.3. PCA, Volcano Plot, Venn Diagram, Heat Map, Protein–Protein Interactions, and Gene Set Enrichment Analysis

PCAGO was applied as a web-based service for principal component analysis (PCA) with a variance cut-off at 500 genes. Zero read counts were removed [31]. For volcano plot generation, we used VolcaNoseR, a web-based tool [32]. The up- and down-regulated genes were used for further analysis under the premise of a minimum log2 2.5-fold change (>5.7-fold change) and a minus log10 significance level of 2.0 (*p* < 0.01) [32]. We also used InteractiVenn: a web-based tool for analyzing sets through Venn diagrams [33]. Heat map analysis was performed with the R interface Morpheus (https://software.broadinstitute.org/morpheus, accessed on 9 December 2024). The 12.0 version of STRING database systematically collects and integrates protein–protein interactions, both physical interactions and functional associations [34]. The G:Profiler version e111_eg58_p18_f463989d was used as a functional enrichment analysis tool that integrates many databases, including gene ontology [35].

## 3. Results

### 3.1. Principal Component Analysis of Gene Expression Changes by Activated THP1 and U937

**Figure 1:**
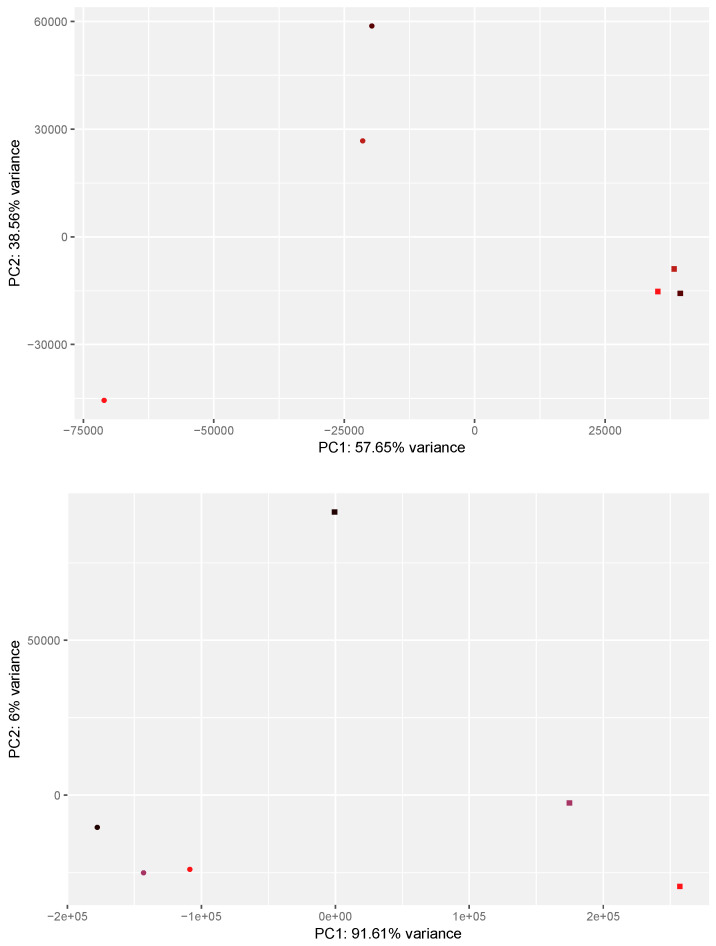
PCA for differentially expressed genes in LPS-IFNG activated THP1 (**up**) and U937 (**down**) cells. Controls and LPS_IFNG are indicated as circles and squares, respectively. Colors represent connected pairs of one experiment. The plot shows the samples projected onto the two-dimensional space spanned by the covariance matrix’s first and second principal components. The expression levels used as input are raw data counts from the most influential 500 genes.

Here, we examined the impact of 24 h LPS and IFNG exposure on the global gene expression profile of THP1 and U937 cells by bulk RNA sequencing. Our preliminary data suggest that 24 h is more effective than 3 h exposure in changing *CXCL8* expression (Supplement Files). Principal component analysis revealed a shift in PC1 caused by exposure to LPS and IFNG in THP1 (57.7%) and U937 (91.6%) cells. In THP1 cells, there was also a considerable shift in PC2 (38.6%) (Figure 1). The variance cut-off at 500 genes is depieced in the Supplementary Files. For THP1 cells, these genes include, apart from cytokines and chemokines, eight cluster of differentiation, sixty-nine ribosomal proteins, eleven heat shock proteins, seven S100 proteins, five NADH dehydrogenase subunits, and six integrin subunits. For U937 cells, also chemokines (*CCL1-5*, *CCL3L3*, *CCL4L2*, and *CCL20*), three cluster of differentiation, sixty-one ribosomal proteins, six heat shock proteins, five S100 proteins, six NADH dehydrogenase subunits, and five integrin subunits were listed. Based on a Venn analysis, THP1 and U937 have 290 genes in common based on PCA (Supplementary Files). The PCA, therefore, already anticipates the massive impact of LPS and IFNG on gene expression changes with similarities but also differences between THP1 and U937 cells (Figure 1).

### 3.2. Analysis of Gene Expression Changes by LPS and IFNG Exposed THP1 and U937 Cells

**Figure 2:**
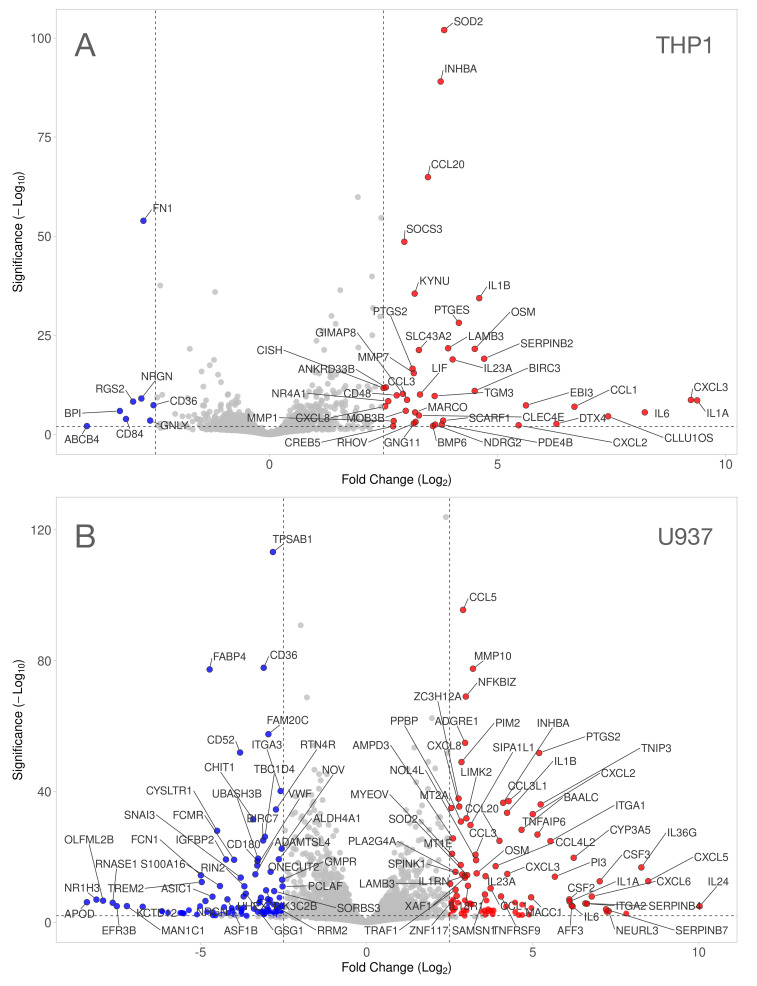
Volcano plot analysis of differentially expressed genes in THP1 and U937 cells treated with LPS and IFNG. Volcano plot analysis identified upregulated (red) and downregulated (blue) genes in (**A**) THP1 and (**B**) U937 cells exposed to LPS and IFNG. The annotated dots are data points with the largest (Manhattan) distance from the origin and are above the thresholds indicated by the dashed line. The threshold was set at 2.0 −log10_padj significance level and 2.5-log2-fold change. While all 51 genes (43 up- and 8 down-regulated genes) are highlighted in the THP1 cells, only the top 100 genes out of 197 genes (94 up- and 103 down-regulated genes) are dyed in the U937 cells.

Next, we performed a volcano analysis to identify changes in our dataset (Figure 2; Supplement Files). Our volcano plot combines a stringent 2.0 −log10 significance level with 2.5-log2-fold change to identify genes that display great magnitude expression changes with a highly statistical significance. When applying these stringent criteria, we could identify 43, 8 up 43, 8 down-regulated genes in THP1 and U937 cells, respectively (Figure 2). Considering that THP1 cells are less responsive than U937, we lowered the threshold to a 1.5-log2-fold change and identified 115 total, 87 up- and 28 down-regulated, genes.

### 3.3. Heat Map of Gene Expression Changes by LPS and IFNG Exposed THP1 and U937 Cells

**Figure 3:**
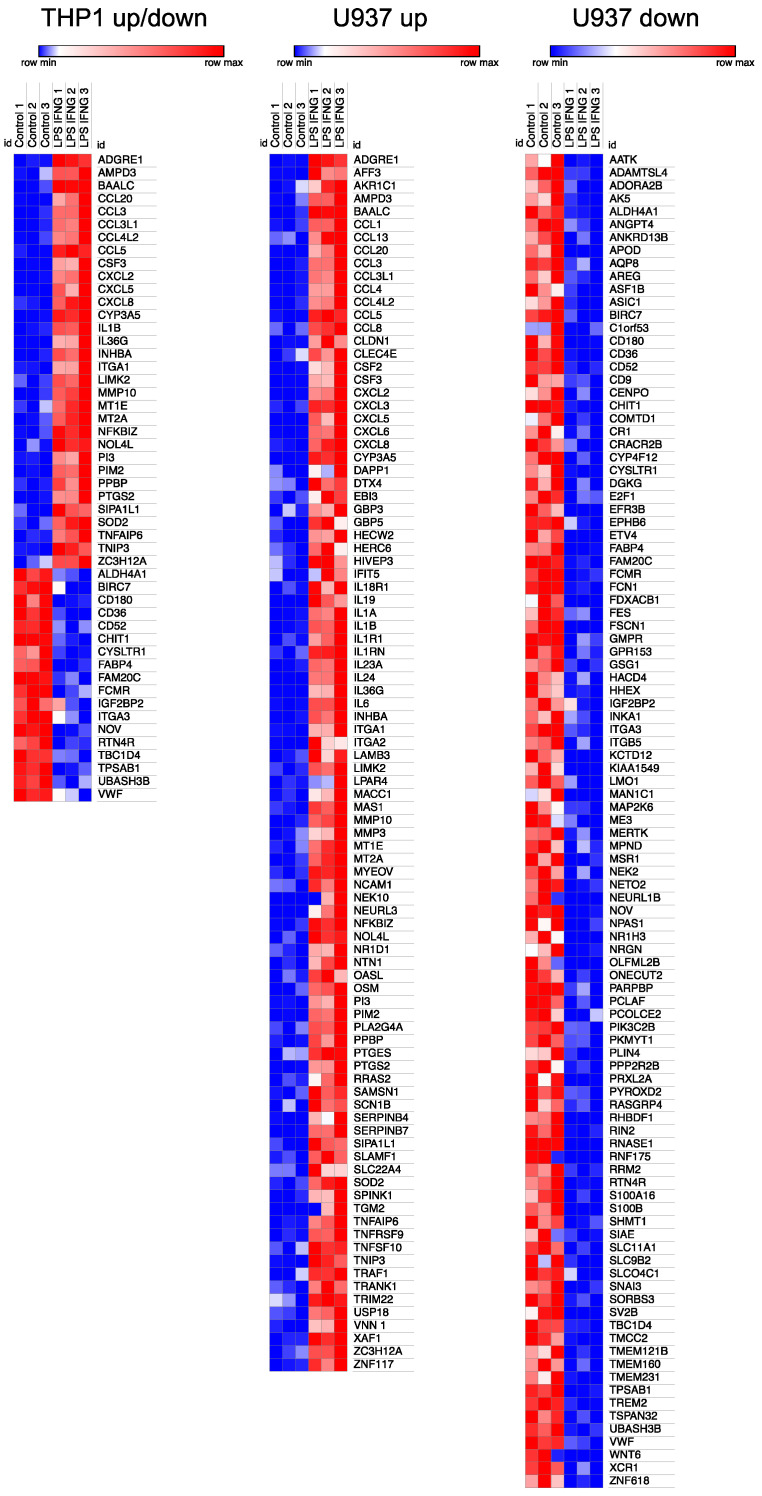
Heat map analysis for differentially expressed genes in THP1 and U937 cells treated with LPS and IFNG. Red specifies high expression levels, while blue shows low levels, indicated by darker and lighter shades of red and blue. Genes with a 2.5-log2-fold change and a significance of 2.0 −log10 were included in this analysis. A relative color scheme uses the minimum and maximum values in each row to convert values to colors.

Next, we performed a heat map analysis of the 51 genes and 197 genes identified with the volcano analysis in THP1 and U937 cells. Figure 3 highlights the variance and magnitude of the differential gene expression. The heat map also displays the lower response of THP1 compared to U937 cells when stimulated with LPS and IFNG (Figure 3).

### 3.4. Analyzing Similarities and Differences Between LPS and IFNG Exposed THP1 and U937 Cells

**Figure 4:**
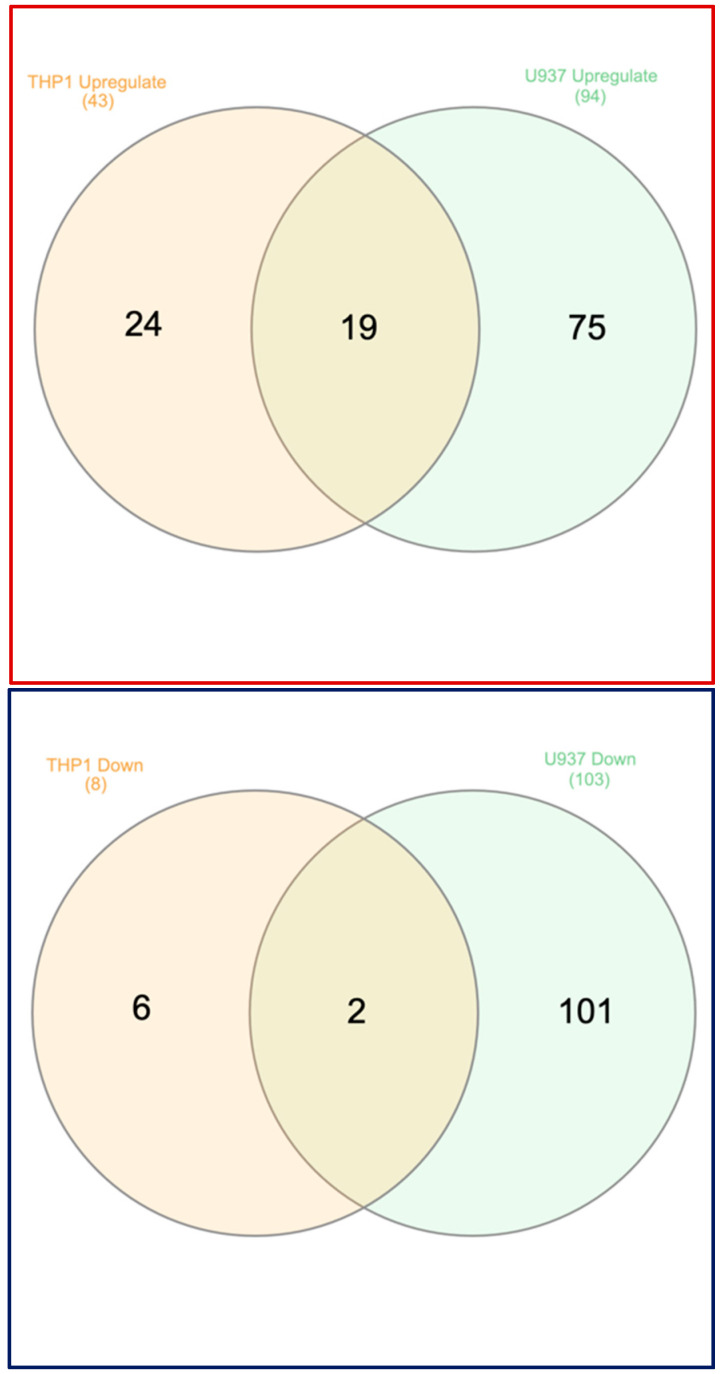
Venn analysis of the genes expressed in THP1 and U937 cells exposed to LPS and IFNG. This analysis is restricted to the genes selected under 2.5-log2-fold change and a significance of 2.0 −log10. Red and blue frames represent up-and-down-regulated genes, respectively. Please note the 19 and 2 commonly up- and down-regulated genes.

Following, a Venn analysis was performed to show the differential response of THP1 and U937 cells to LPS and IFNG stimulation (Figure 4; Supplement Files). Venn analysis revealed 19 commonly upregulated genes (*CCL1*, *CCL3*, *CCL20*, *CXCL2*, *CXCL3*, *CXCL8*, *CLEC4E*, *DTX4*, *EBI3*, *IL1A*, *IL1B*, *IL23A*, *IL6*, *INHBA*, *LAMB3*, *OSM*, *PTGES*, *PTGS2*, *SOD2*) and 2 downregulated genes (*NRGN* and *CD36*). Moreover, 24 and 75 genes were upregulated and 6 and 101 were downregulated, independently, considering the stringent threshold applied in THP1 and U937 cells, respectively. When decreasing the threshold of THP1 cells to 1.5 log2 changes, only 5 additional common up-regulated genes were identified: *S100A16*, *ZC3H12A*, *NCAM1*, *PYROXD2*, *SIPA1L1*.

### 3.5. STRING Analysis of Expression Changes in THP1 and U937 Cells Exposed to LPS and IFNG

**Figure 5:**
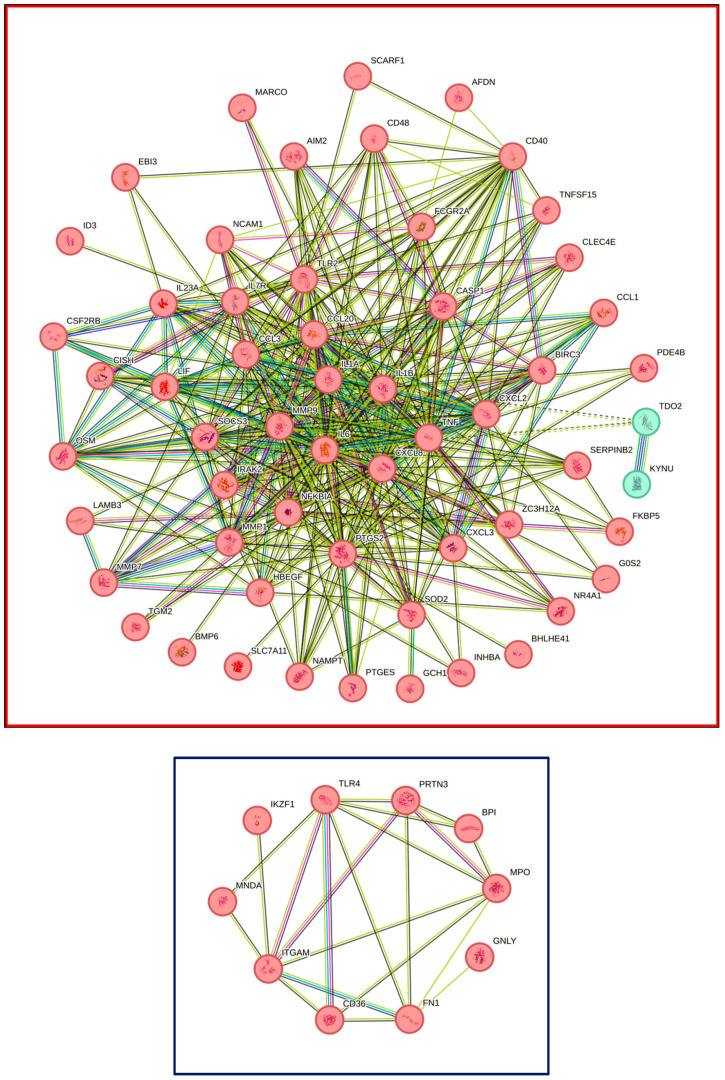
STRING analysis of up-and down-regulated genes in THP1 cells exposed to LPS and IFNG. The protein-protein association network and functional enrichment analyses of the 51 genes with differential expression are shown. Red and blue frames represent the STRING analysis of 43 up- and 8 down-regulated genes, respectively.

The STRING analysis was performed to identify clusters of all 51 regulated genes in THP1 cells (Figure 5). This analysis revealed one major cluster based on 30 up-regulated genes linked to inflammation, including cytokines (*IL1A*, *IL1B*, *IL23A*, *IL6*, *LIF*), chemokines (*CCL1*, *CCL3*, *CCL20*, *CXCL2*, *CXCL3*, *CXCL8)*, and *PTGES* and *PTGS2*, also including *BMP6*, *MMP1*, *MMP7*, and *SOD2* (red frame; Supplementary Files). Only one cluster of down-regulated genes, including *TLR4*, *ITGAM*, *MPO*, *FN1*, and *CD36*, was observed (blue frame; Supplementary Files).

**Figure 6:**
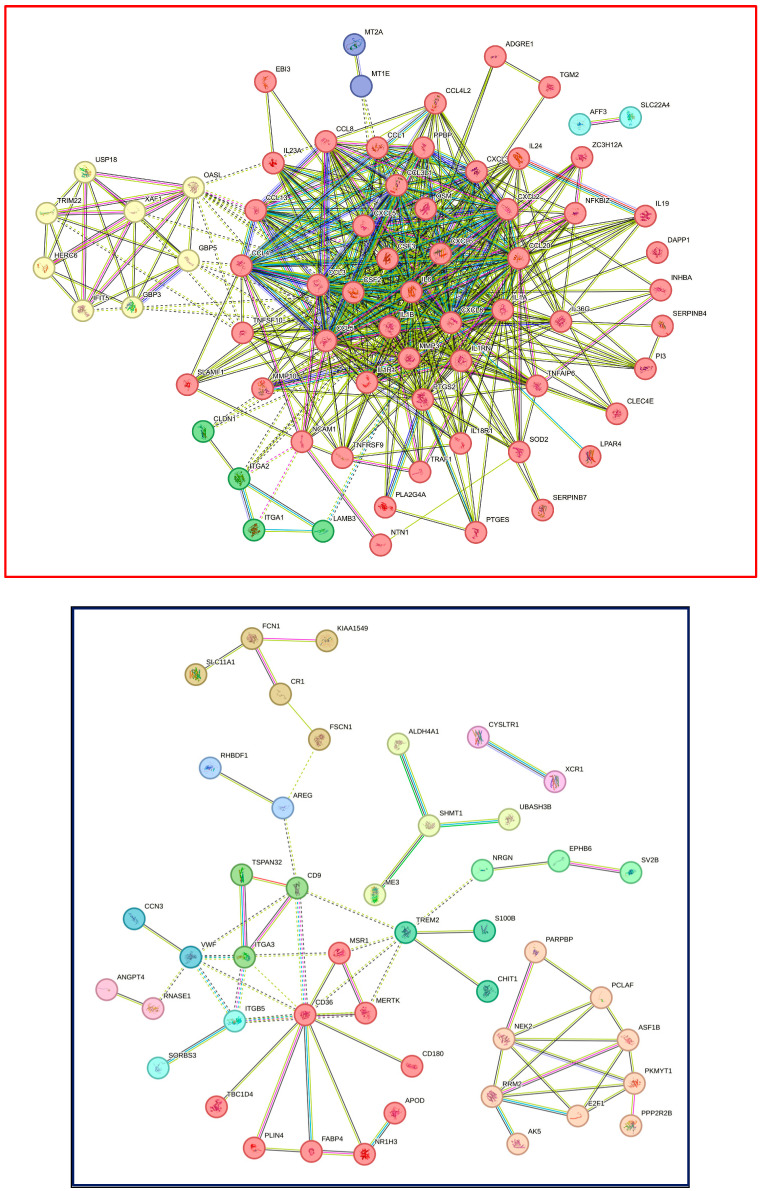
STRING analysis of up- and down-regulated genes in U937 cells exposed to LPS and IFNG. The protein–protein association network and functional enrichment analyses of the 197 genes with differential expression are shown. Red and blue frames represent the STRING analysis of 94 up- and 103 down-regulated genes, respectively.

STRING analysis of U937 caused a more complex picture of up-regulated genes with a total of 5 clusters, including on the cluster interaction with the cytokine and the cytokine receptor, similar to the THP1 cells, but more complex with cytokines (*IL1A*, *IL1B*, *IL1R1*, *IL1RN*, *IL6*, *IL18R1*, *IL19*, *IL23A*, *IL24*, *IL36G*,) and chemokines *(CCL1*, *CCL3*, *CCL3L1*, *CCL4*, *CCL4L2*, *CCL5*, *CCL8*, *CCL13*, *CCL20*, *CXCL2*, *CXCL3*, *CXCL5*, *CXCL6*, *CXCL8)*, *PTGES* and *PTGS2*, and *MMP3*, *MMP10*—and a series of other clusters highlighting *CSF2*, *CSF3*, *ITGA1* and *ITGA2*, as well as *MT1E* and *MT2A* (Figure 6). Down-regulated genes produce even 12 clusters containing *CD180* and *CD36*, *CD9* and *ITGA3*, or *CHIT1*, *S100B*, *TREM2*. The complete list of clusters related to THP1 and U937 cells is in the Supplement Files. This STRING analysis demonstrates the similarities and differences in the clustering of genes, not only showing a more complex response of U937 cells over THP1 cells but also highlighting the complexity of the down-regulated genes.

### 3.6. G:Profiler Analysis of Gene Expression Changes in THP1 and U937 Cells by LPS and IFNG

**Figure 7:**
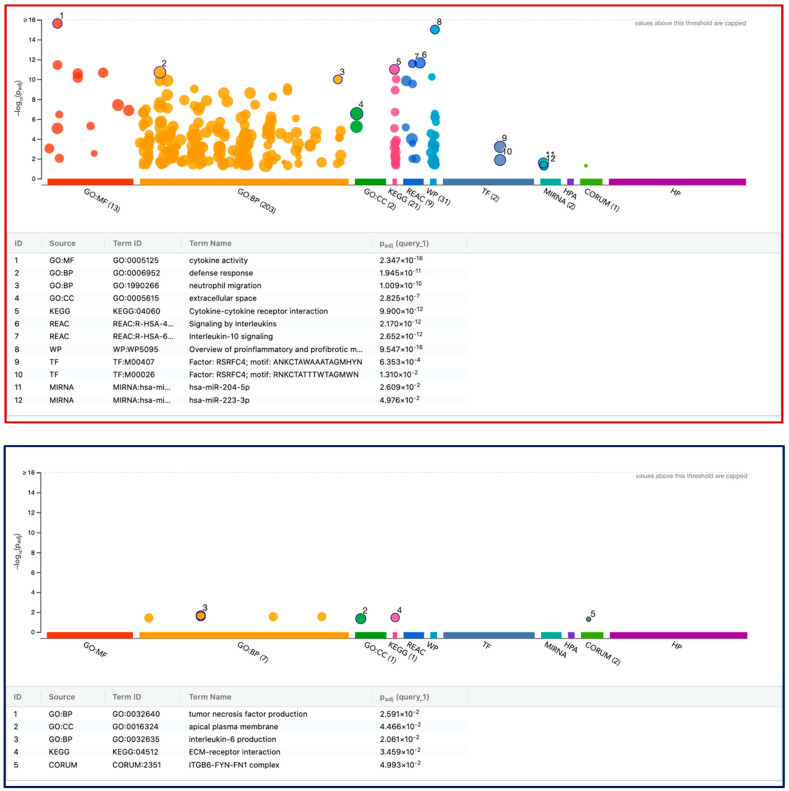
G:Profiler analysis of differentially expressed genes in THP1 with LPS and IFNG. Functional enrichment analysis, also known as over-representation analysis (ORA) or gene set enrichment analysis, was performed using the G:Profiler online tool. The red and blue frames represent the STRING analysis of 43 up- and 8 down-regulated genes, respectively. Selected top significant pathways were highlighted and labeled numerically. Using the Benjamini–Hochberg method, the *p*-value was adjusted (Padj) for multiple testing.

We further performed a functional enrichment analysis of the strongly regulated 51 genes (43 up- and 8 down-regulated genes) in THP1 cells (Figure 7, Supplement Files). Consistent with the STRING clustering analysis, in THP1 cells, the G:Profiler analysis revealed highly significant enrichment of upregulated genes that are linked to cytokine activity (GO:0005125), defense response (GO:0006952), and cytokine–cytokine receptor interaction (KEGG:04060). The transcription factor RSRFC4 (T.F.:M00407, T.F.:M00026) and two miRNAs, hsa-miR-204-5p and hsa-miR-223-3p, were identified. ECM–receptor interaction (KEGG:04512) clusters with only *FN1* and *CD36* among the downregulated genes.

**Figure 8:**
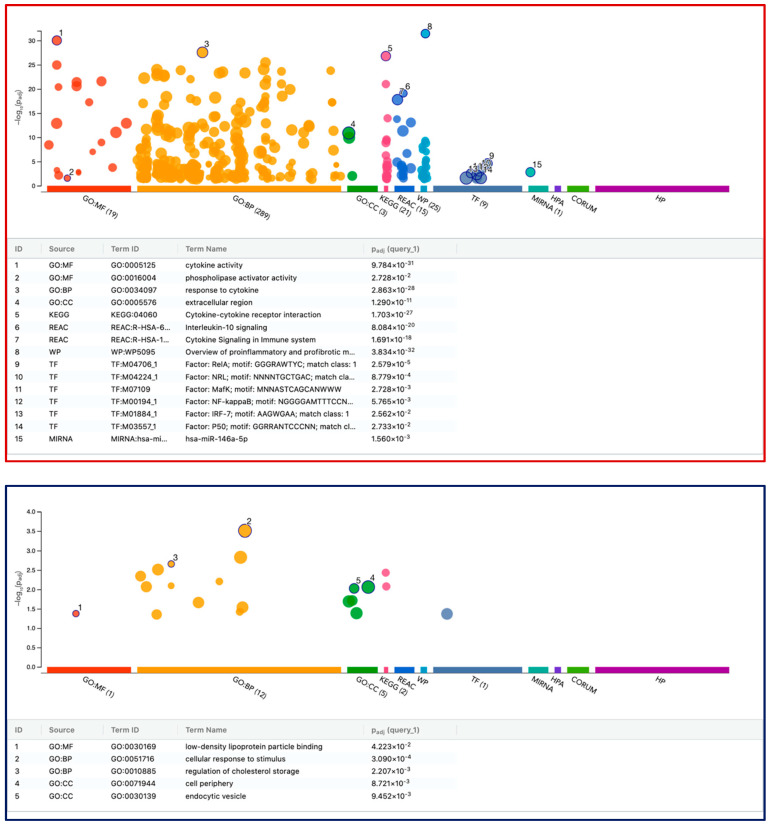
G:Profiler analysis of differentially expressed genes in U937 with LPS and IFNG. Functional enrichment analysis, also known as over-representation analysis (ORA) or gene set enrichment analysis, was performed using the G:Profiler online tool. The red and blue frames represent the analysis of 94 up- and 103 down-regulated genes, respectively. Selected top significant pathways were highlighted and labeled numerically. Using the Benjamini–Hochberg method, the *p* value was adjusted (Padj) for multiple tests.

In U937 cells, 197 genes (94 up- and 103 down-regulated genes) differentially expressed upon exposure to LPS and IFNG (Figure 8). As anticipated from STRING analysis, G:Profiler showed highly significant enrichment of upregulated genes related to cytokine activity (GO:0005125), response to cytokine (GO:0034097), cytokine–cytokine receptor interaction (KEGG:04060), and a series of transcription factors, including *RelA, NRL, MafK, NF-kappaB, IRF-7*, and *P50*, as well as the *miR-146a-5p*. The downregulated genes were clustering in the G:Profiler; for instance, in G.O.: M.F. low-density lipoprotein particle binding (GO:0030169), it was CD36, MSR1, and TREM2. And in GO:BP cellular response to stimulus (GO:0051716), the list is long, with 59 genes, including *CD9, CD36, CD180*, as well as *ITGA3, ITGB5, S100B, TREM2, CR1, VWF, APOD, AREG FCN1, FABP4, CCN3*, *WNT6*, and *IGFBP2.*

### 3.7. RT-PCR Gene Expression Changes in THP1 and U937 Cells by LPS and IFNG

**Figure 9:**
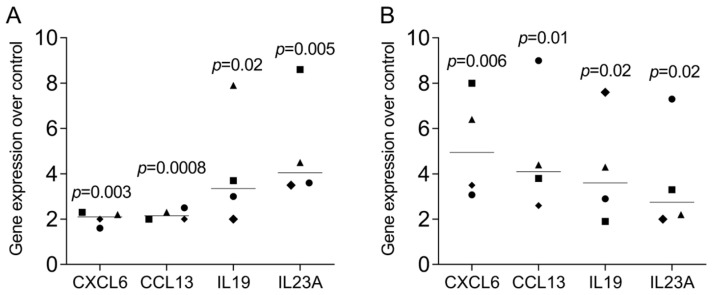
RT-PCR analysis of selected genes in (**A**) THP1 and (**B**) U937 cells exposed to LPS and IFNG. Cells were exposed to LPS and IFNG for 24h before RT-PCR analysis was performed. Statistical analysis was based on data from four independent experiments using ratio-paired t-test with untreated cells serving as controls.

Finally, to confirm the findings of the RNAseq analysis, we have selected a panel of genes that are not commonly known to be expressed in THP1 and U937 cells to be verified by gene expression analysis in independent experiments. RT-PCR analysis confirms the observations from the RNAseq screening approach (Figure 9), even though the expression levels were comparable lower.

## 4. Discussions

About 16,000 and 14,000 hints appearing in PubMed support the global use of THP1 and U937 cell lines in experimental research. The reasons for working with these cell lines are the easy availability, the comparison of findings, the reproducibility of the data, avoiding donor variability, and the drawbacks of preparing the monocytes. It was a logical consequence, therefore, not only to use the two cell lines but also to compare their responses in bioassays by primary [21,22] and secondary intension [23,24]—similar to what has been performed with transcriptional profiling of macrophages derived from monocytes and iPS cells [36]. The response of THP1 [17,18] and U937 [19,20] to a combination of LPS and IFNG was used to initiate an inflammatory response. However, even though the inflammatory response was identified by expression analysis of individual marker genes—mostly cytokines and chemokines—the global signature of the two cell lines remained unclear. Thus, the opportunity to use the cell lines to study inflammatory responses, and to use this knowledge to establish new and refine existing bioassays, deserves refinement. The present research is, therefore, a primer to identify new highly regulated target genes in THP1 and U937 cells, not only limited to those being upregulated but also downregulated genes.

If we relate our findings to those of others, we can acknowledge RNAseq or gene assays approaches to characterize the response to inflammatory clues on macrophage cell lines. For instance, genome array screening with U937 cells under LPS activation was performed [37]. The same is true for adherence-induced differentiation of primary human monocytes into macrophages, which examined the in vitro transcriptional profile [38] and expression changes in CDV-11-infected DH82 cells, a macrophage-derived cell line from canines [39]. This research has paved the way for an approach to compare the response of THP1 and U937 cells to an LPS and IFNG induction at the bulk RNAseq level. We also appreciate the innovation of single-cell RNA sequencing. This advanced RNAseq strategy revealed the dynamic heterogeneity of U937 cells in response to LPS [40], and a paracrine dependency in the LPS response of U937 cells was proposed [41]. Similar attempts with LPS-treated THP-1 cells showed that *IL8* and *IL1B* were expressed in 84 and 63% of cells together with enrichment of PPAR and HIF-1α signaling pathways, and an anti-inflammatory M2-like state characterized by the expression of *NR3C1, JAK2*, and *IRAK3* [42]. In addition, RNAseq of murine RAW264.7 macrophages and bone marrow macrophages were reported [43]. Thus, taking the opportunity of single-cell RNAseq into account, future research can confirm our findings based on bulk RNAseq and characterize the cell response’s heterogeneity in the THP1 and U937 cells at the single-cell level.

One significant finding was that THP1 cells show a weaker response than U937 cells when exposed to LPS and IFNG, indicated by the 43, 8 up- and 94, 103 down-regulated genes in THP1 and U937 cells, respectively. This list of genes was defined by the stringent criteria of a 2.5-log2-fold change and a significance of 2.0 −log10. Apart from the sensitivity, the response per se was also differential; only 19 and 2 genes were commonly expressed under this condition. Moreover, considering that THP1 cells are less sensitive, we have performed another analysis with a 1.5-log2-fold change at the same significance level (115 genes total), and 87 up- and 28 down-regulated genes were identified. Under these less stringent conditions, the Venn analysis revealed another 5 common genes, suggesting that only a small number of shared genes can be grouped into a gene panel, for instance, to use both cell lines to test the response to biomaterials or other potential agonists and antagonists of an inflammatory response. A practical gene panel could involve testing for a modulation of a pro-inflammatory response might include a set of chemokines (*CCL1*, *CCL3*, *CCL20*, *CXCL2*, *CXCL3*, *CXCL8*), cytokines (*IL1A*, *IL1B*, *IL23A*, *IL6*) prostaglandin synthetases (*PTGES*, *PTGS2*) and perhaps *CD36* to have downregulated genes that can also be detected by flow cytometry. This panel of inflammatory mediators is further reflected by the GO analysis genes highlighting cytokine activity, defense response, and cytokine–cytokine receptor interaction.

U937 cells showed a complex response to LPS and IFNG activation, which offers more opportunities to assemble a gene panel for a bioassay. The gene panel of up-regulated genes can be extended for other cytokines not commonly expressed (*IL1R1*, *IL1RN*, *IL18R1*, *IL19*, *IL24*, *IL36G*) and (*CCL3L1*, *CCL4*, *CCL4L2*, *CCL5*, *CCL8*, *CCL13*, *CXCL5*, *CXCL6*). The list of chemokines is partially identical to LPS-exposed primary macrophages and iPS-derived cells, but it exceeds our list for *CCL18*, *CCL19*, *CCL7*, and *CXCL11* [36]. The complex response of U937 is represented by the GO analysis with the enrichment of genes related to cytokine activity and cytokine–cytokine receptor interaction. Other candidates for establishing a bioassay might include the colony-stimulating factors CSF2 and CSF3 that bind to hemopoietic stem cells and promote the production of white blood cells, and integrin subunits α1 (*ITGA1*) and α2 (*ITGA2*), as well as metallothionein-1E and -2 (MT1E and MT2A). Other than THP1 cells, U937 cells enables us to establish a panel of downregulated genes that is, however, heterogenous and allows flow cytometry of *CD9, CD36* and *CD180*, and also gene expression on *ITGA3*, *CHIT1*, *S100B*, and *TREM2*, depending on the research question. Gene panels can be assembled based on the cluster analysis. Our finding suggests a heterogenicity of the THP1 and U937 cells in their expression changes as 24 and 75 genes were upregulated, and 6 and 101 were downregulated, independently, in THP1 and U937 cells. Please note the 19 and 2 commonly up- and down-regulated genes.

We further consider the study limitation of our research. Once we have a comparative setting and RNAseq analysis, the data should only be extrapolated to reflect the response of primary macrophages to LPS and IFNG. Nevertheless, our data and future research can further uncover to which extent the THP1 and U937 cells represent the response of the primary macrophages and which parts of the genetic signature are limited to the cell line and vice versa. It would also be worth understanding how differentiating the cells with PMA affects the overall response to LPS and IFNG, comparing the floating with the adherent THP1 and U937 cells. We should state that we have not performed an extensive validation of the RNAseq data using RT-PCR or proteomics methods, and the expression changes we have observed with RT-PCR of the selected genes were less than impressive. It thus needs another round of theoretical and practical screening to propose a robust bioassay with all its ingredients, such as primer lists or protein-based analysis. Nevertheless, as bulk RNAseq becomes more accessible, future bioassays may be based on this technology and perhaps even replace downstream protocols based on traditional single gene expression analysis. Moreover, RNAseq can be performed with proteomics of the culture medium and the cell lysates to refine today’s analytical spectrum of bioassays. Another limitation and, at the same time, inspiration for future research is the use of other agonists than LPS and IFNG, for instance, specific agonists of individual pattern recognition receptors or even direct exposure to microbiota, and the screening for pharmacological and natural compounds capable of modulating the inflammatory response. Perhaps it is worth studying the expression changes on the single cell level; even though THP1 and U937 are of clonal origin, their response to LPS and IFNG could be synchronized. Thus, plenty of study limitations exist, but all these considerations pave the way for future research.

In conclusion, we have completed an RNAseq comparison of THP1 and U937 exposed to LPS and IFNG. Our study suggests that THP1 macrophages are potentially less responsive to exploring immune responses than the U937 monocytic cells. Our findings will broaden the spectrum of applications of THP1 and U937 cells for bioassay research and, in particular, add to the list of potential target genes primarily but not exclusively related to inflammatory research. The present study may serve as a primer in bioassay research, ultimately replacing the traditional approaches based on selected gene expression by bulk RNAseq.

## Data Availability

Supplement Files hold the analysis of PCA, Volcano blots, Venn diagram, String analysis clustering, and G:Profiler, respectively. DESeq2results and raw_gene_counts are also available in the Supplement Files.

## Supplementary Materials

The following supporting information can be downloaded at: https://www.mdpi.com/article/10.3390/cells13242062/s1.
