# Supplementary material for: Inflammatory Response of THP1 and U937 Cells: The RNAseq Approach
Source: Cells. 2024 Dec 13;13(24):2062. doi: 10.3390/cells13242062 (PMC11674919; doi:10.3390/cells13242062)
Supplement: Supplementary file 1 [file cells-13-02062-s001.zip › Supplement Files PCA.zip!/PCA blot.zip!/ THP1 PCA plot.pdf]

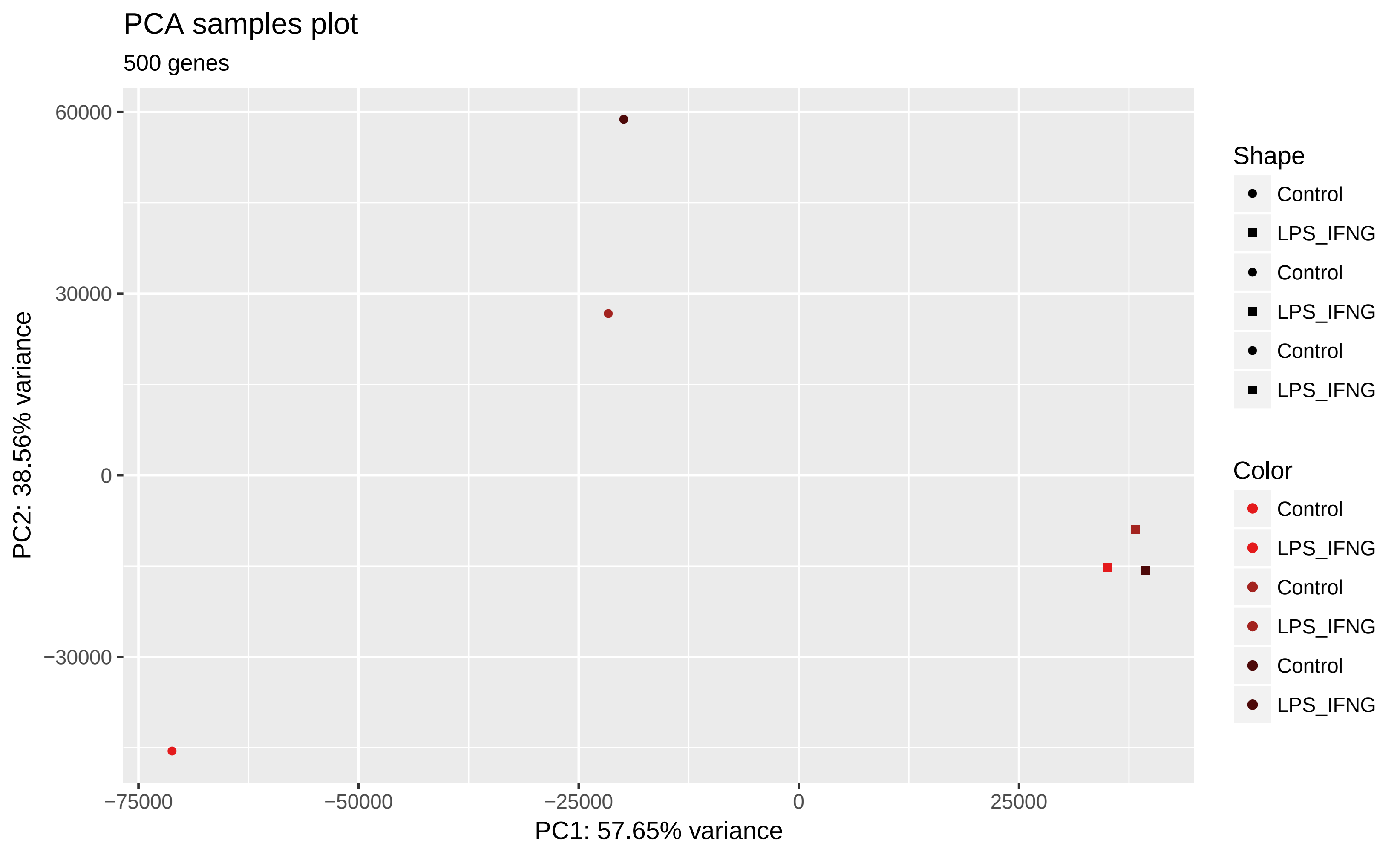
PCA samples plot
500 genes
60000
30000
0
−30000
−75000
−50000
−25000
0
25000
PC1: 57.65% variance
PC2: 38.56% variance
Shape
Control
LPS_IFNG
Control
LPS_IFNG
Control
LPS_IFNG
Color
Control
LPS_IFNG
Control
LPS_IFNG
Control
LPS_IFNG
