# Supplementary material for: Inflammatory Response of THP1 and U937 Cells: The RNAseq Approach
Source: Cells. 2024 Dec 13;13(24):2062. doi: 10.3390/cells13242062 (PMC11674919; doi:10.3390/cells13242062)
Supplement: Supplementary file 1 [file cells-13-02062-s001.zip › Supplement Files PCA.zip!/PCA blot.zip!/U937 PCA plot.pdf]

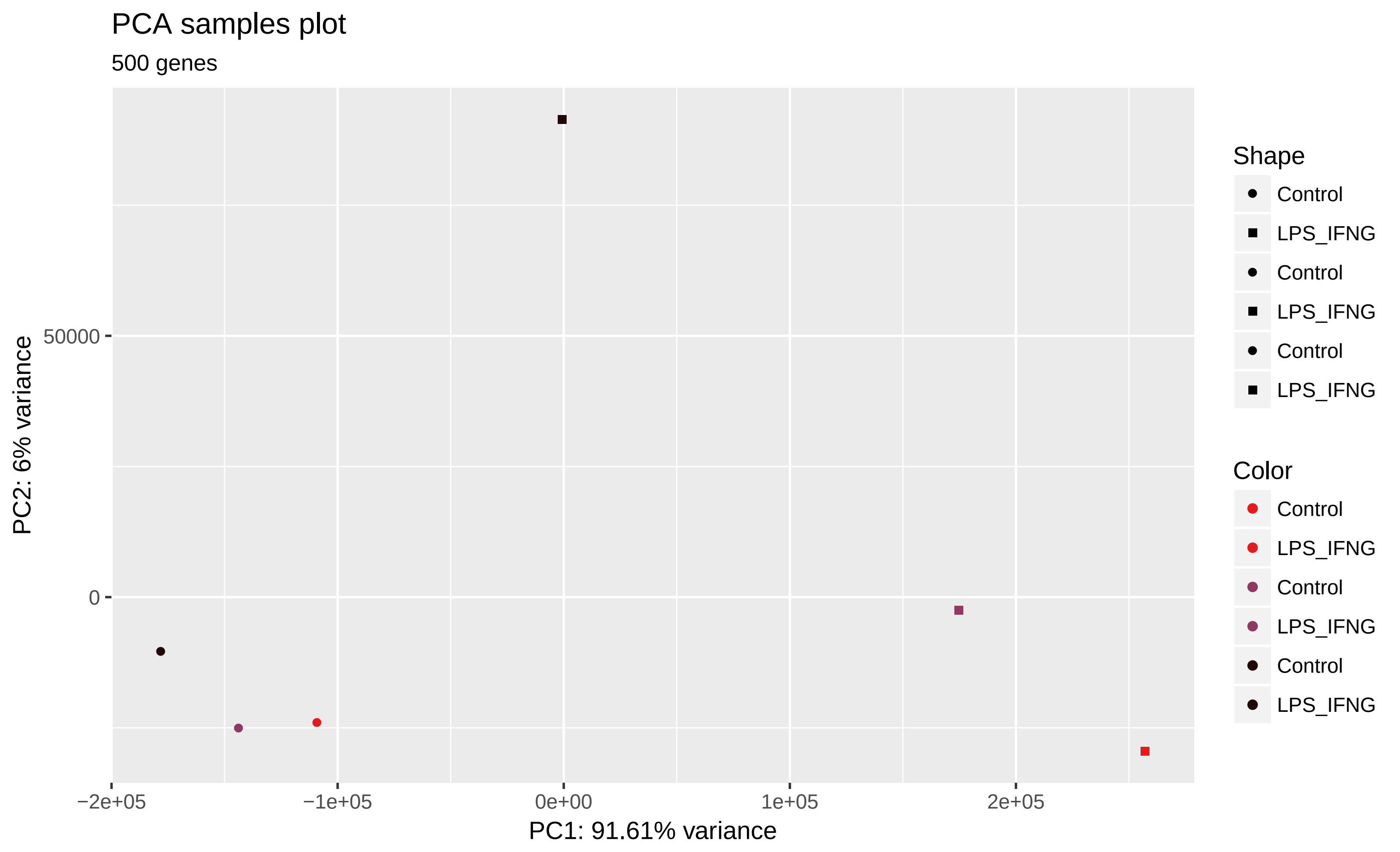

PCA samples plot
500 genes
50000
0
−2e+05
−1e+05
0e+00
1e+05
2e+05
PC1: 91.61% variance
PC2: 6% variance
Shape
Control
LPS_IFNG
Control
LPS_IFNG
Control
LPS_IFNG
Color
Control
LPS_IFNG
Control
LPS_IFNG
Control
LPS_IFNG
